# Total alkaloids of bulbus of *Fritillaria cirrhosa* alleviate bleomycin-induced inflammation and pulmonary fibrosis in rats by inhibiting TGF-β and NF-κB signaling pathway

**DOI:** 10.29219/fnr.v67.10292

**Published:** 2023-12-29

**Authors:** Mingxin Pai, AGA Er-bu, Yexin Wu, Tse Wai Ming, Tse Kathy Wai Gaun, Bengui Ye

**Affiliations:** 1 Key Laboratory of Drug-Targeting and Drug Delivery System of the Education Ministry, Sichuan Engineering Laboratory for Plant-Sourced Drug and Sichuan Research Center for Drug Precision Industrial Technology, West China School of Pharmacy Sichuan University, Chengdu, China; 2 School of Medicine, Tibet University, Lasa, China; 3 Nin Jiom Medicine Manufactory (H.K.) Limited, Hong Kong, China

**Keywords:** Fritillaria cirrhosa, total alkaloids, pulmonary fibrosis, inflammation, TGF-*β*, NF-*κ*B

## Abstract

**Background:**

Bulbus of *Fritillaria cirrhosa* is a medicinal and edible plant that has the functions of clearing away heat and moisturizing the lungs, resolving phlegm, and relieving coughs. Its ethanol extract has been proven to have a therapeutic effect on lung diseases. Pulmonary fibrosis is a respiratory disease that forms scars in lung tissue, leading to severe respiratory problems. However, the therapeutic effect of total alkaloids of bulbus of *Fritillaria cirrhosa* (BFC-TA) on pulmonary fibrosis has not been confirmed.

**Objective:**

This study aimed to investigate the therapeutic effect of total alkaloids of *Fritillaria cirrhosa* on pulmonary fibrosis rat model and explore its potential mechanism.

**Design:**

The total alkaloids in the bulbus of *Fritillaria cirrhosa* were purified using cation exchange resin. The alkaloids contained in the BFC-TA were identified, and the concentration of alkaloids was determined by High Performance Liquid Chromatography-Diode Array Detector-Evaporative Light Scattering Detector (HPLC-DAD-ELSD). Bleomycin (BLM) (5.0 mg/kg) was instilled into the trachea of 60 rats to establish a pulmonary fibrosis model. After 7 days, BFC-TA (34.2, 68.4, and 136.8 mg/kg) was administered continuously for 21 days. During this period, the body weight changes of the rats were measured, the levels of hydroxyproline (HYP) and inflammatory factors were measured in the collected serum, and the histological analysis of the lung tissue was performed by staining technology. Western blotting and quantitative Polymerase Chain Reaction (qPCR) were used to assess the protein and gene composition of inflammation and transforming growth factor-β (TGF-β) signaling pathways.

**Results:**

Nine main components (Peimisine, Imperialine-3-β-D-glucoside, Yibeinoside A, Imperialine, Peiminine, Isopeimine, Hupehenine, Delavinone, Ebeiedinone) were determined by HPLC-DAD-ELSD, and the contents of Peimisine, Imperialine-3-β-D-glucoside and Imperialine were determined. BFC-TA (34.2, 68.4, and 136.8 mg/kg) reduced the levels of pro-inflammatory factors, increased the levels of anti-inflammatory factors, dose-dependently improved the morphology of lung tissue. And during epithelial-mesenchymal transition process, BFC-TA dose-dependently reduced the expression of E-cadherin, dose-dependently increased the expression of Fibronectin. In addition, Western blot analysis and qPCR results showed that inhibiting NF-κB and TGF-β-related signaling pathways effectively slowed down the occurrence of BLM-induced pulmonary fibrosis in rats. And the therapeutic effect of BFC-TA (136.8 mg/kg) is better than that of pirfenidon (PFD) (150 mg/kg).

**Conclusion:**

BFC-TA effectively alleviates the progression of the BLM-induced pulmonary fibrosis rat model by regulating the inflammatory response in the lungs and the expression of the TGF-β signaling pathway.

## Popular scientific summary

- This is the first report exploring the therapeutic effect of total alkaloids of bulbus of *Fritillaria cirrhosa* on bleomycin-induced pulmonary fibrosis in rats.
- Total alkaloids of bulbus of *Fritillaria cirrhosa* can reduce the degree of pulmonary fibrosis and inflammation in rats with bleomycin-induced pulmonary fibrosis.
- Total alkaloids of bulbus of *Fritillaria cirrhosa* inhibit the inflammatory response by regulating NF-κB signaling pathway and delay the process of pulmonary fibrosis by regulating TGF-β signaling pathway.

A perennial herb in the Liliaceae family, bulbus of *Fritillariae cirrhosa* (BFC), is a member of the Fritillaria genus. BFC is a kind of traditional Chinese medicine that has been given official approval for use as food as well as medication. There are now 42 different types of functional foods containing fritillaria in the register, including two that include the bulbus of *Fritillariae cirrhosa*, which is mostly used to control immunity, clean the throat, and moisten the lungs (1). It can moisturize and cool the lung, disperse mucus and ease coughing, remove anthracome and stagnation, and eliminate heat from the body (2). Alkaloids, polysaccharides, sterols, volatile oils, and other chemical components are among those found in BFC. The primary active components of BFC are thought to be alkaloids (3–5). The ethanol extract of BFC has been shown in several earlier studies to have a protective effect against pulmonary fibrosis (6), and the major active component of BFC, total alkaloids of bulbus of *Fritillaria cirrhosa* (BFC-TA), has significant scientific implications.

A chronic, life-threatening interstitial lung illness known as pulmonary fibrosis is defined by histology and/or chest high-resolution CT (HRCT) characteristics for prevalent interstitial pneumonia (UIP). The elderly are easily affected, and the reason is unclear (7). Multiple, ongoing genetic, and/or environmental causes may damage the lung tissue and eventually result in pulmonary fibrosis. The cells go through a mesenchymal-like transformation, exhibiting a pro-fibrotic phenotype, secreting a lot of pro-fibrotic factors, developing a pro-fibrotic microenvironment, activating fibroblasts into myofibroblasts, and building up an excessive amount of extracellular matrix (ECM) (8, 9). These modifications lead to structural damage to the lungs as well as loss of function. A water-soluble glycopeptide antibiotic with anti-tumor qualities is bleomycin. It comes from a collection of glycopeptides that have been determined from the bacterium Streptomyces (10). Bleomycin is a toxin that can destroy tumor cells; nevertheless administering it may negatively impact individuals in ways that are permanent. Lung toxicity is one of the most significant adverse effects and might result in treatment-dependent lung fibrosis. The cause is that bleomycin cannot be broken down by an enzyme present in lung tissue (11). Transforming growth factor-β (TGF-β) is a multifunctional protein that has the ability to control a wide range of cell processes, including cell division, proliferation, and apoptosis in several organ systems. Both the etiology of lung illness and normal lung morphogenesis and function depend on TGF-β (12). It regulates the propagation of fibroblasts to the site of tissue damage and mediates fibrosis by preventing alveolar epithelial cell growth and apoptosis or by promoting fibroblast differentiation into myofibroblasts, producing ECM proteins, and preventing ECM degradation (13).

Pirfenidone and nintedanib are the major medications used to treat pulmonary fibrosis in clinical settings today. These medications have been authorized by the Food and Drug Administration (FDA) (14). However, the therapy can only relieve symptoms and cannot stop the progression of pulmonary fibrosis. More adverse responses are present (15, 16), and the mortality rate is still significant. BFC-TA is a natural remedy extracted from plants that has a modest impact and few adverse effects. It has potential for use in pulmonary fibrosis research.

## Material and methods

### Preparation of BFC-TA

BFC were gathered from the Nin Jiom Medicine Manufactory’s standardized bulb planting base in Haidong City, Qinghai Province, China. It was identified by the Department of Pharmacognosy, West China College of Pharmacy, Sichuan University. The West China College of Pharmacy at Sichuan University’s Laboratory of Pharmacognosy deposited the voucher specimens.

Reflux extraction with 70% ethanol was used to get the ethanol extract of BFC, which was subsequently isolated and purified. In conclusion, it has successfully produced an early-stage ethanol extract of BFC (30.31% yield, weight for weight of the raw material). The insoluble material was eliminated when the dark brown ethanol extract was treated in 2–4% hydrochloric acid and filtered. The pH was adjusted to 1–4 using the saturated sodium hydroxide solution, and the resin and raw medicinal ingredients were put in the column in a 4:1:1:5 ratio. The sample solution with adjusted pH was loaded onto the treated 001 × 7 cation exchange resin, and the sample was repeatedly loaded. After the sample was loaded, the gradient elution was performed at a flow rate of 2–6 BV/h according to the following steps: (1) Elution of 6–10 BV with water, (2) 5–12 BV was eluted with 60% ethanol solution of 5% sodium chloride, and (3) The 60% ethanol solution elution part of 5% sodium chloride was collected. The BFC-TA (45% yield, weight for weight of the raw material) were obtained after the collected elution fraction was concentrated, desalted, and dried.

Dissolve 35 mg of BFC-TA in 5 mL of chromatographic methanol to prepare a 7 mg/mL test solution for the determination of the three alkaloid contents in BFC-TA. Dissolve 40 mg of BFC-TA in 2 mL of chromatographic methanol to prepare a 20 mg/mL test solution for the identification of nine alkaloids in BFC-TA.

### Determination of content of BFC-TA

The acid dye colorimetry combined with the UV-visible spectrophotometer (TU-1810SPC, Beijing Purkinje General Instrument Co., ltd, China) was used to determine the content of BFC-TA according to the ‘content determination method’ described in the first section of the Chinese Pharmacopoeia (Chinese Pharmacopoeia Commission, 2020 Edition).

### Determination of three alkaloids in BFC-TA and characterization of nine alkaloids in BFC-TA by HPLC-DAD-ELSD

The alkaloids in BFC-TA were analyzed with a gradient elution HPLC. A Thermo Scientific Ultimate 3,000 high performance liquid chromatograph (Thermo Scientific, USA) was used to determine it. Venusil XBP C18 chromatographic column (250 mm × 4.6 mm, 5 μm) was used, 0.03% diethylamine (A)-acetonitrile (B) was used as mobile phase, elution gradient (0–10 min, 30% B; 10–35 min, 30% → 60% B; 35–45 min, 60% B; 45–65 min, 60% → 90% B; 65–75 min, 90% B), column temperature 25 °C, flow rate 1 mL min^-1^, injection volume 10 µL for the determination of the three alkaloid contents, injection volume 25 µL for the identification of nine alkaloids; using SEDERE SEDEX 90 evaporative light scattering detector, gasification temperature 40°C, air pressure 3.5 bar.

### Animals

Male Sprague-Dawley rats (SPF grade, 6 weeks old, weighing 200 ± 20 g certificate number: SCXK (Sichuan) 2020-030) were purchased from Chengdu Dashuo Experimental Animal Co., Ltd., China and raised in a ventilated cage in an air-conditioned room with a temperature of 22 ± 2°C and a humidity of 65 ± 5°C. The 12-h light-dark cycle. Rats were adaptively fed for 1 week before the experiment. All animal operations were authorized by Sichuan University’s Institutional Animal Ethics Committee (IAEC) and completely adhered to Chinese law for the use and care of laboratory animals (SYXK (Chuan) 2018-113).

### Bleomycin-induced pulmonary fibrosis model

**Fig. 1:**
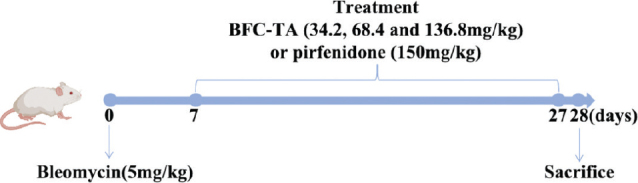
SD rat models of Bleomycin-induced pulmonary fibrosis and treatment with BFC-TA and PFD

Six groups made up of 72 male Sprague-Dawley rats were created at random: negative control (NC) group (*n* = 12 rats per group), model group (*n* = 12 rats per group), BFC-TA treatment group (34.2, 68.4 and 136.8 mg/kg, *n* = 12 rats per group), positive control (PFD) group (150 mg/kg). Pentobarbital sodium (45 mg/kg) was administered intraperitoneally to all of the rats to make them unconscious. All of the rats received a single intratracheal injection of 5.0 mg/kg BLM to cause pulmonary fibrosis, except the NC group. The rats in the NC group (control group) received the same dosage of regular saline. On the seventh day after BLM induction, rats in the total alkaloid group and the positive control group were administered orally with pirfenidone and total alkaloid dissolved in normal saline. The rats in the NC group and the model group both got the same quantity of normal saline over the course of 21 days. Record rats body weight per 3 days. The experimental set up is shown in Fig. 1.

### Animal tissue collection and processing

After 28 days, SD rats were anesthetized with 1% pentobarbital sodium (5 mg/kg). After bleeding from the abdominal aorta, the whole lung was rapidly detached, taken out, and weighed. The right lung was quickly frozen in liquid nitrogen and used for the measurement of other indicators. The remaining tissues and organs were frozen for 48 h in 4% paraformaldehyde (BL539A; Biosharp, China). The right-sized tissue samples were chosen for standard paraffin embedding and sectioning. Hematoxylin-eosin (H&E) and Masson trichrome staining, together with an optical microscope (CX33RTFS2; image courtesy of Olympus Corporation, Japan), were used to investigate the tissue morphology and alterations. The calculation formula of lung coefficient is as follows:


$$\text{Lung coefficient }=\frac{\text{Lung weight (mg)}}{\text{Body weight (mg)}}\times 100\%$$

### Measurement of hydroxyproline (HYP) assay

Alkaline hydrolysis was used to calculate the HYP concentration. The following method is based on the HYP test kit (# A030-2-1; Nanjing Jianchen Bioengineering Institute, China). A test tube containing 50 mg of freshly cut right lung tissue and 1 mL of hydrolysate was used. The test tube was then heated for 20 min in a water bath to facilitate the hydrolysis reaction. The pH level of the pyrolysis liquid was adjusted to between 6.0 and 6.8 once it had reached room temperature. After centrifuging the lysate at 7,000 g for 10 min at room temperature, 1 mL of the supernatant was transferred to a new test tube. Each sample’s absorbance at 550 nm was measured using an ultraviolet-visible spectrophotometer (TU-1810SPC, Beijing Purkinje General Instrument Co., Ltd., China), and the findings were compared to the HYP standard curve.

### Enzyme-linked immunosorbent assay (ELISA)

SD rats were put unconscious by an intraperitoneal injection of 1% sodium pentobarbital (5 mg/kg). A disposable blood collection needle was used to draw 5 mL of blood into a vacuum blood collection tube that contained anticoagulant after the abdominal cavity had been opened to show the abdominal aorta. A chilled centrifuge (TGL-16S, Sichuan Shuke Instrument Co., Ltd., China) was used to spin blood at a speed of 6,000 g for 20 min at 4°C. The levels of TNF-α, IL-1, IL-6, IL-18, IL-4, and IL-10 secretion in rat serum were determined using ELISA kits. 50 mg of fresh right lung were homogenized with 1 mL of PBS using a high-speed low-temperature tissue grinder (KZ-III-FP, Wuhan Sevier Biotechnology Co., Ltd., China; Gibco, MA, USA). After centrifuging the homogenate at 6,000 g for 15 min at 4°C, the supernatant was separated into samples. The steps were followed precisely as directed by the ELISA kit. The ELISA kits were provided by the Wuhan Geneme Biotechnology Co., Ltd. business in China. The absorbance was determined at 450 nm using a microplate reader (K3 TOUCH, Thermo Fisher Scientific, USA). Cytokine content is shown in pg/mL.

### Western blotting analysis

A tissue lysate containing protease and phosphatase inhibitors (#BR006, Signalway Antibody, College Park, MD, USA) and phenylmethylsulfonyl fluoride (PMSF, #ST506, Beyotime, China) solution (P0013B, Shanghai Biyuntian Biotechnology Co., Ltd., China) was added to 50 mg of fresh right lung, and the supernatant was then taken. In order to determine the protein content, bicinchoninic acid (BCA protein test kit, #P0010, Beyotime, China) was utilized. An equivalent amount of 50 g of protein was loaded onto a 7.5% sodium dodecyl sulfate-polyacrylamide gel (SDS-PAGE gel; #PG111, Epizyme, China) before being transferred to a polyvinylidene fluoride (PVDF) membrane (#IPVH00010, Millipore, Billerica, MA, USA). Membranes were blocked in Tris-buffered saline Tween-20 (TBST) buffer containing 5% (wt/vol) nonfat dry milk for 1 h at room temperature. Following that, the membrane was incubated with a number of primary antibodies for an entire night at 4°C. GAPDH served as the internal control. The membrane was washed with TBST solution and then incubated with a secondary antibody that was HRP labeled for an hour at room temperature. The protein bands were subsequently seen using an enhanced chemiluminescence detection method (Fig. S5).

Antibody: Collagen I (ab60043, Abcam); alpha smooth muscle Actin (ab124964, Abcam); IκBα (ab32518, Abcam); p-Smad2 (ab280888, Abcam); NF-κB (CST/4764S, cell signaling technology); p-NF-κB (CST/3033S, cell signaling technology); Smad2 (12570-1-AP, proteintech); Fibronectin (CST/26836S, cell signaling technology); E-Cadherin (CST/3195T, cell signaling technology); GAPDH (11F7886, Affinity Biosciences).

### RNA extraction and quantitative real-time polymerase chain reaction

About 200 μL of TRIzol reagent (R1006, Beyotime, China) was used to extract total RNA. In a 20 μL reaction, 1 μg of total RNA was converted into cDNA using the PrimeScriptTMRT reagent Kit with gDNA Eraser (RR047A, TaKaRa, Japan) for subsequent real-time qPCR tests. To run real-time qPCR on the QuantStudio system (Thermo Fisher Scientific, Waltham, MA, USA), follow the manufacturer’s instructions and use the SYBRPRIME PCR KIT (Fast HS) kit (BG0014, Bioground, China). Each reaction was performed three times (Table S1).

### Statistical analysis

GraphPad Prism 9 (GraphPad Software Inc., USA) was used for all statistical analysis. The information was shown using mean ± standard deviation. Two-way repeated measures analysis of variance (ANOVA) and a post hoc test were used to assess weight data. With regard to the other data, one-way ANOVA and subsequent Tukey post hoc tests were used to assess group differences. *P* < 0.05 was used to determine statistical significance.

## Results

### Content of BFC-TA

Standard curve was used to determine the total alkaloid content of BFC-TA (Fig. S3). The results indicated that BFC-TA has a total alkaloid concentration of 45.54 ± 0.03%. By using HPLC-DAD-ELSD, the three alkaloids that make up the total amount of alkaloids in BFC were examined. Table S2 provides a summary of the three alkaloids and the overall number of alkaloids in BFC.

The HPLC-DAD-ELSD spectrum of the BFC-TA was shown in Fig. S2. According to the standard curve of the three alkaloids prepared by the external standard method, the contents of Peimisine, Imperialine-3β-D-glucoside, and and Imperialine in the BFC-TA were calculated to be 36.09 ± 0.07 mg/g, 81.70 ± 0.35 mg/g, and 193.37 ± 0.22 mg/g, respectively (Fig. S4).

### Qualification of nine alkaloids in BFC-TA

**Fig. 2:**
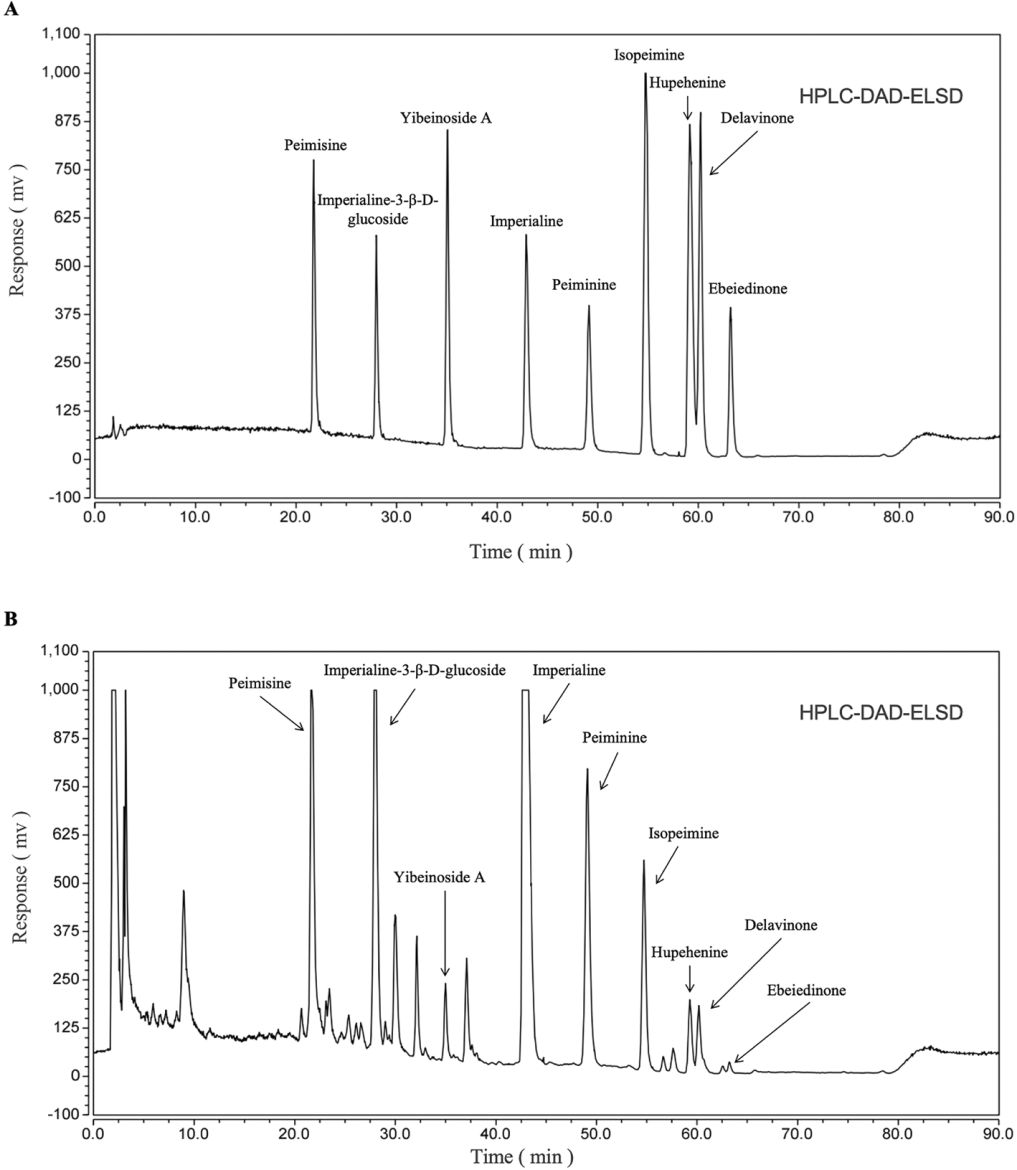
HPLC-DAD-ELSD spectrum of BFC-TA (Increased concentration and loading volume) and nine isosteroidal alkaloids of BFC-TA. (A) Mixed solution of nine alkaloid standards; (B) HPLC-DAD-ELSD analysis of BFC-TA (Increased concentration and loading volume).

In previous studies, alkaloids in BFC have been isolated and identified (17), and we characterized nine alkaloids (Peimisine, Imperialine-3-β-D-glucoside, Yibeinoside A, Imperialine, Peiminine, Isopeimine, Hupehenine, Delavinone, Ebeiedinone) (Table 1). Larger sample concentrations and injection volumes cause flat peaks, leading to errors in quantitative results. In addition, the smaller sample concentration and injection volume cause many peaks in BFC-TA to fail to appear. Therefore, in order to characterize the alkaloids in BFC-TA, the sample concentration (20 mg/mL) and injection volume (20 mL) were increased, and the results are shown in Fig. 2.

**Table 1:** Identification of isolated from BFC-TA characteristic peaks

Retention Time (min)	Fractions	CAS
21.733	Peimisine	19773-24-1
27.982	Imperialine-3-β-D-glucoside	67968-40-5
35.073	Yibeinoside A	98985-24-1
42.833	Imperialine	61825-98-7
49.123	Peiminine	18059-10-4
54.730	Isopeimine	23496-43-7
59.155	Hupehenine	98243-57-3
60.217	Delavinone	96997-98-7
63.210	Ebeiedinone	25650-68-4

BFC-TA, total alkaloids of bulbus of *Fritillaria cirrhosa*.

### The BFC-TA reduce bleomycin-induced lung injury

**Fig. 3:**
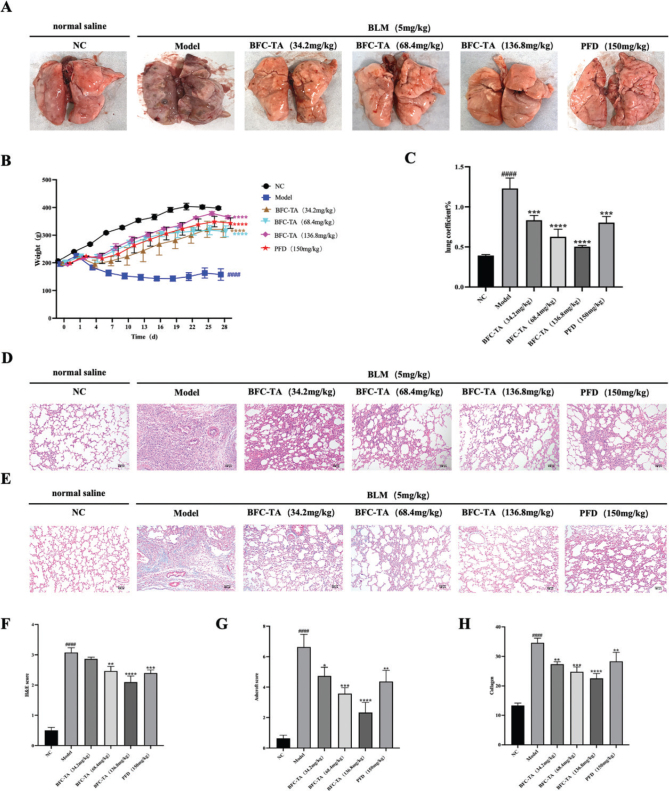
The effect of total alkaloids bulbus of *Fritillaria cirrhosa* (BFC-TA) on the appearance of lung tissue, body weight, lung coefficient and lung histopathology of rats. (A) Observation of the appearance of lung tissue after 28 days of sacrifice; (B) Body weight was recorded every 3 days after modeling; (C) Lung coefficient = (lung weight (g))⁄(body weight (g)) × 100%; (D) Representative images of hematoxylin-eosin (H&E) staining. Scale bar: 100 μm; (E) Representative images of Masson’s trichrome staining. Scale bar: 100 μm; (F) HE stain score; (G) Masson staining Ashcroft score; (F) image j quantification of Masson staining collagen fibers. Data are presented as mean ± standard deviation (*n* = 12). ^####^*P* < 0.0001 versus the NC group. *P < 0.05, **P < 0.01, ***P < 0.001, ****P < 0.0001 versus the BLM group.

To verify the efficacy of BFC-TA on pulmonary fibrosis, we observed the changes of physiological indexes of rats treated with BLM for 28 days and the pathological changes of lung tissue after 28 days of treatment. On the 8th day after intratracheal BLM treatment, different concentrations of BFC-TA were administered by gavage, and pirfenidone was used as a positive control group. As shown in Fig. 3A, the appearance of lung tissue in the NC group was pink, uniform in color, soft in texture, smooth in surface, no spots, and other pathological changes. The surface of lung tissue in the Model group was pale or dark red, with poor gloss, the unclear outline of lung lobe, hard texture, blunt edge, poor elasticity, scattered bleeding points, and ecchymosis. After treatment with BFC-TA and pirfenidone, the color of lung tissue and the changes of cord-like grooves and nodules in rats were improved to varying degrees. The body weight of the rats in the model group was much lower after receiving BLM therapy than the NC group, as can be shown in Fig. 3B-C, which was a key marker of how severe the pathological state was. This impact was dose dependently diminished by BFC-TA. After 28 days of execution, the BFC-TA reduced the lung coefficient of rats in a dose-dependent manner, and the rats treated with high doses of BFC-TA were significantly better than those treated with pirfenidone. The pulmonary fibrosis of SD rats was examined by HE staining and Masson staining (Fig.3D–E), which showed the pathological changes of lung tissue in rats. Compared with the NC group, BLM treatment resulted in alveolar structure destruction, inflammatory cell infiltration, alveolar septum thickening, collagen fiber hyperplasia, and the BFC-TA reduced the pathological changes of lung tissue in a dose-dependent manner. HE score, Ashcroft score, and Image J quantified collagen fibers in Masson-stained lung tissue (Fig. 3F–H) showed that the degree of pulmonary fibrosis was significantly lower than that of the model group after administration of BFC-TA, and showed a better therapeutic effect than the pirfenidone treatment group.

### The BFC-TA inhibit TGF-*β*-induced *ε*pithelial-mesenchymal transition (EMT) process

**Fig. 4:**
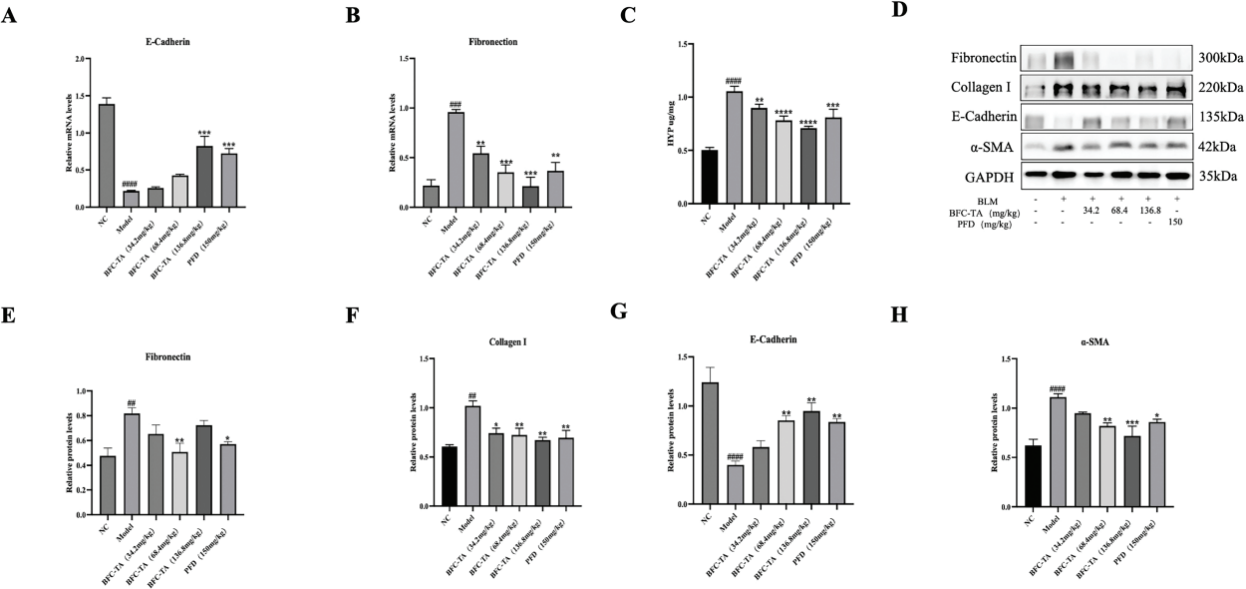
The effect of total alkaloids bulbus of *Fritillaria cirrhosa* (BFC-TA) on levels of E-cadherin mRNA, α-SMA, COL-1 protein and HYP content in rat lung tissue. (A) The mRNA expression levels of E-cadherin in lung tissue by qPCR; (B) The mRNA expression levels of Fibronectin in lung tissue by qPCR; (C) The content of HYP in lung tissue; (D) The protein exression levels of Fibronectin, E-cadherin, Collagen I and α-SMA in lung tissue were analyzed by Western blotting; (E) Quantitative analysis of protein expression levels of Fibronectin by Western blotting. Relative ratios of the indicated protein expression were normalized to GADPH and expressed as mean values; (F) Quantitative analysis of protein expression levels of Collagen I by Western blotting; (G) Quantitative analysis of protein expression levels of E-cadherin by Western blotting; (H) Quantitative analysis of protein expression levels of E-cadherin by Western blotting. Western blotting and qPCR were repeated at least 3 times (*n* = 3). And only a representative Western blotting result was placed in Fig. 4D. Data are presented as mean ± standard deviation. ^####^*P* < 0.0001, ^###^*P* < 0.001, ^##^*P* < 0.01 versus the NC group. *P < 0.05, **P < 0.01, ***P < 0.001, ****P < 0.0001 versus the BLM group.

To determine whether the BFC-TA can inhibit pulmonary fibrosis after BLM administration through EMT reaction, we measured the expression of E-cadherin and fibronectin in epithelial cells. The expression of α-SMA, Collagen I and HYP (the primary component of collagen) was measured in mesenchymal cells after 28 days for the EMT process generates a large amount of ECM and TGF-β promotes the transformation of lung fibroblasts into myofibroblasts and generates a large amount of α-SMA (Fig. 4A–E). The results showed that in BLM-induced rats, Fibronectin, α-SMA, Collagen I, and HYP were significantly upregulated, and E-Cadherin was significantly downregulated. After treatment with BFC-TA, the expression of α-SMA, COL-1, and HYP was downregulated in a dose-dependent manner, and the protein expression levels of E-Cadherin were upregulated. Due to the low content of fibronectin protein, different doses of BFC-TA had no obvious trend on its inhibitory effect. In addition, the protein content of E-Cadherin can also be seen in Fig. 4A, so we detected the mRNA content of fibronectin. From Fig. 4G-H, bleomycin-induced rat fibronectin was significantly upregulated, and E-Cadherin was significantly upregulated. Following BFC-TA treatment, the expression of fibronectin was dose-dependently downregulated, whereas the expression of E-Cadherin was dose-dependently upregulated. This indicates that BFC-TA, characterized by the EMT process, reduces the BLM-induced pulmonary fibrosis process.

### The BFC-TA reduce bleomycin-induced inflammation

**Fig. 5:**
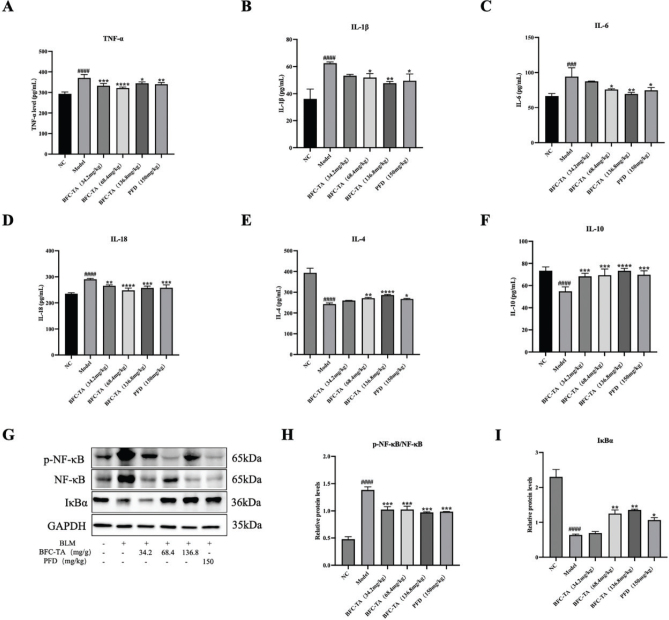
The effect of total alkaloids bulbus of *Fritillaria cirrhosa* (BFC-TA) on pro-inflammatory factors, anti-inflammatory factors in rat serum and NF-κB signaling pathway proteins in lung tissue. (A) Serum TNF-α concentration was determined by Elisa; (B) Serum IL-1β concentration was determined by Elisa kit; (C) Serum IL-6 concentration was determined by Elisa kit; (D) Serum IL-18 concentration was determined by Elisa kit; (E) Serum IL-4 concentration was determined by Elisa kit; (F) serum IL-10 concentration determined by Elisa kit; (G) The protein expression levels of p-NF-κB, NF-κB and IκBα in lung tissue were analyzed by Western blotting; (H) Quantitative analysis of protein expression levels of p-NF-κB/NF-κB by Western blotting. Relative ratios of the indicated protein expressions were normalized to GADPH and expressed as mean values; (I) Quantitative analysis of protein expression levels of IκBα by Western blotting. Serum data are presented as mean ± standard deviation (*n* = 12). Western blotting were repeated at least 3 times (*n* = 3), and only a representative result in Fig. 5G. Data are presented as mean ± standard deviation. ^####^*P* < 0.0001, ^###^*P* < 0.001 versus the NC group. *P < 0.05, **P < 0.01, ***P < 0.001, ****P < 0.0001 versus the BLM group.

As the rats treated with BLM were characterized by inflammation in the first 7 days, the inflammatory factors and inflammatory signaling pathways were studied to evaluate the effect of BFC-TA. As seen in Fig. 5A–F and Table 2, the model group dramatically lowered the secretion level of anti-inflammatory factors (IL-4, IL-10) and raised the secretion level of pro-inflammatory factors (TNF-α, IL-1, IL-18, and IL-6) in the serum of rats treated with BLM. TNF-α, IL-1, IL-18, and IL-6 secretion levels in the blood of BLM-treated rats were dramatically reduced after BFC-TA administration, but IL-4 and IL-10 secretion levels were significantly elevated. The NF-κB signaling pathway is an inflammation-related signaling pathway. The effect of BFC-TA on NF-κB signaling pathway in the treatment of pulmonary fibrosis was further studied (Fig. 5G–H). Compared with the NC group, the ratio of p-NF-κB/NF-κB in the model group was significantly increased, and the expression level of NF-κB pathway inhibitor protein IκBα was significantly decreased. The BFC-TA reduced the ratio of p-NF-κB/NF-κB in a dose-dependent manner and increased the expression level of IκBα protein, indicating that the BFC-TA inhibited the activation of NF-κB signaling pathway induced by BLM.

**Table 2:** *Serum* figure of rats in each groups

Groups	TNF-(α (pg/mL)	IL-1β (pg/mL)	IL-6 (pg/mL)	IL-18 (pg/mL)	IL-4 (pg/mL)	IL-10 (pg/mL)
NC	300.00 ± 8.84	36.18 ± 5.85	66.57 ± 2.96	235.21 ± 3.17	393.81 ± 37.63	73.47 ± 2.98
Model	370.31 ± 15.27^####^	62.49 ± 0.70^####^	94.28 ± 10.15^###^	290.56 ± 2.69^####^	242.81 ± 9.93^####^	54.83 ± 3.45^####^
BFC-TA(34.2 mg/kg)	332.950 ± 10.01^***^	53.22 ± 0.83	87.37 ± 0.36	265.55 ± 2.76^**^	260.17 ± 3.32	68.33 ± 2.36^***^
BFC-TA(68.4 mg/kg)	321.44 ± 5.03^****^	51.91 ± 2.32^*^	75.87 ± 0.87^*^	248.18 ± 7.15^****^	271.51 ± 9.60^**^	69.37 ± 4.84^***^
BFC-TA(136.8 mg/kg)	344.65 ± 5.19^*^	47.70 ± 1.05^**^	69.62 ± 1.74^**^	257.35 ± 5.63^***^	286.27 ± 10.01^****^	73.34 ± 1.87^****^
PFD(150 mg/kg)	339.97 ± 6.86^**^	49.57 ± 4.04^*^	74.71 ± 3.17^*^	258.12 ± 8.91^***^	267.53 ± 8.02^*^	69.77 ± 3.05^***^

All data are presented as mean ± standard deviation (*n* = 12).

#### *P* < 0.0001,

### *P* < 0.001 versus the NC group.

* *P* < 0.05,

** *P* < 0.01,

*** *P* < 0.001,

**** *P* < 0.0001 versus the BLM group. BFC-TA, total alkaloids of bulbus of *Fritillaria cirrhosa*.

### The BFC-TA inhibit TGF-*β*/Smad signaling in rats treated with BLM

**Fig. 6:**
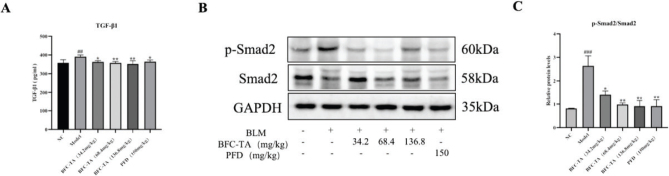
The effect of total alkaloids bulbus of *Fritillaria cirrhosa* (BFC-TA) on transforming growth factor-β (TGF-β)/Smad signaling pathways in rats. (A) Elisa kit was used to determine the content of TGF-β1 in rat serum; (B) The protein expression levels of p-Smad2 and Smad2 in lung tissue were analyzed by Western blotting; (C) Quantitative analysis of protein expression of p-Smad2/Smad2 by Western blotting. Relative ratios of the indicated protein expressions were normalized to GADPH and expressed as mean values. Serum data are presented as mean ± standard deviation (*n* = 12). Western blotting were repeated at least 3 times (*n* = 3), and only a representative result in Fig. 6B. Data are presented as mean ± standard deviation. ^###^*P* < 0.001, ^##^*P* < 0.01 versus the NC group. *P < 0.05, **P < 0.01 versus the BLM group.

As a major factor in the development of pulmonary fibrosis, TGF-β may slow down the progression of the condition by preventing the signaling pathway from becoming activated. The key protein in the Smad signaling pathway is Smad2, and the Smad signaling pathway is the downstream signaling pathway of TGF-β. Therefore, by examining the TGF-β/Smad signaling pathway, we looked into the function of BFC-TA in the therapy of pulmonary fibrosis. The findings demonstrated that the model group’s TGF-β1 (Fig. 6A) and p-Smad2/Smad2 ratio (Fig. 6B–C) were considerably upregulated compared to the NC group. TGF-β1 and p-Smad2/Smad2 were both markedly and dose-dependently downregulated following BFC-TA therapy. The outcomes demonstrated that BFC-TA prevented the induction of the TGF-β/Smad signaling pathway.

### The BFC-TA inhibit STAT3 signaling in rats treated with BLM

**Fig. 7:**
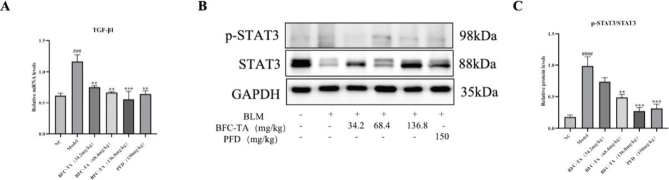
The effect of total alkaloids bulbus of *Fritillaria cirrhosa* (BFC-TA) on STAT3 signaling pathways in rats. (A) The mRNA expression levels of transforming growth factor-β1 (TGF-β1) in lung tissue by qPCR; (B) The protein expression levels of p-STAT3 and STAT3 in lung tissue were analyzed by Western blotting; (C) Quantitative analysis of protein expression of p-Smad2/Smad2 by Western blotting. Relative ratios of the indicated protein expressions were normalized to GADPH and expressed as mean values. Western blotting and qPCR were repeated at least 3 times (*n* = 3). And only a representative Western blotting result was placed in Fig.7B. Data are presented as mean ± standard deviation. ^####^*P* < 0.0001, ^###^*P* < 0.01 versus the NC group. **P < 0.01, ***P < 0.001 versus the BLM group.

The Smad pathway and non-Smad pathway are two downstream signaling pathways that are started when TGF-β binds to receptors. Because the STAT3 signaling route is a Smad-independent signaling pathway, it can be used to further our understanding of the function of BFC-TA in the management of pulmonary fibrosis. The findings demonstrated that the model group’s TGF-β1 and p-STAT3/STAT3 ratios, as well as the expression levels of both molecules, were considerably upregulated compared to the NC group. TGF-β1 and p-STAT3/STAT3 were both markedly and dose-dependently downregulated after BFC-TA therapy (Fig. 7). The findings demonstrated that BFC-TA prevented the stimulation of BLM of the STAT3 signaling pathway.

## Discussion

BFC is a plant that may be used as a functional food and is both edible and therapeutic. It can be used to eliminate heat from the body, moisten the lungs, relieve coughing, and dissolve phlegm. The main effective components of BFC are isosteroidal alkaloids and steroidal alkaloids (18). Previous studies have shown that BFC ethanol extract can protect against pulmonary fibrosis by inhibiting the activation of the NF-κB signaling pathway (6). In addition, studies have shown that some of the monomeric alkaloid components contained in BFC-TA have also been proven to delay the progression of lung-related diseases through multiple pathways, such as COPD and acute lung injury (19, 20). Total alkaloids are the main active ingredients in BFC (21, 22), so it is important to explore whether BFC-TA can all the progression of pulmonary fibrosis.

Bleomycin (BLM) is a multicomponent compound antibiotic of alkaline glycopeptides produced by Streptomyces verticillioides. It has the effect of antitumor, but one of its side effects is pulmonary fibrosis (23). Although bleomycin can effectively kill tumor cells as a toxin, its use can cause irreversible damage to patients. One of the most important side effects is pulmonary toxicity, which may lead to treatment dose-dependent pulmonary fibrosis. The reason is that there exists no enzyme in the lung tissue that can break down bleomycin (11). In this study, tracheal instillation of BLM to the lungs of rats was used. This method has been demonstrated to be the most effective animal model for characterizing an animal model of pulmonary fibrosis that may be utilized for preclinical testing (24). This study found that BLM treatment significantly reduced the body weight of rats and increased the ratio of lung to body in rats. Lung histopathological results showed that BLM treatment could lead to alveolar destruction, inflammatory cell infiltration, and collagen fiber deposition. This indicates that BLM causes damage to lung tissue and alveolar epithelial cells.

There are two phases to the BLM-induced pulmonary fibrosis process: the first step is the inflammatory stage. At the lesion, inflammatory cells gather, and chemokines attract inflammatory cells and fibroblasts to the wounded regions to activate cytokines and growth factors. The second stage is the pulmonary fibrosis stage. Interstitial cell proliferation and matrix collagen aggregation replace normal lung tissue structure (25, 26). Studies have shown that the presence of myofibroblasts in fibroblast lesions is one of the histopathological markers of pulmonary fibrosis, which is induced by many chemokines, the most common of which is TGF-β (27, 28). TGF-β, a multipurpose protein, has three subtypes: TGF-β1, TGF-β2, and TGF-β3, with TGF-β1 being the most prevalent subtype. TGF-β1 releases and binds to the TGF-β type II receptor (TβRII), but TGF-β1 cannot bind to the TGF-β type I receptor (TβRI). TGF-β1 needs to be involved with TβRII to activate TβRI kinase, leading to activate downstream Smad2 and Smad3 protein and cause phosphorylation of Smad2 and Smad3 protein. The phosphorylated Smad2 and Smad3 recruit Smad4 to form a complex, which is transferred to the nucleus to regulate the transcription of TGF-β target genes and output signals (29–32). TGF-β/Smad signaling pathway activation occurs at this point. The STAT3 signaling pathway is a Smad-independent signaling pathway (33). Cytokines (interleukins, interferons, etc.) bind to interleukin receptors and/or interferon receptors on the membrane of lung fibroblast cells and dimerize receptors, resulting in phosphorylation of JAK. Phosphorylated JAK interacts with TGF-β1 to recruit STAT3 and phosphorylate STAT3. Phosphorylated STAT3 forms a dimer, which is transferred to the nucleus, regulates transcription, and outputs signals (34, 35). At this time, the STAT3 signaling pathway is activated. Cytokines (interleukin, TNF-α) bind to TNF receptor 1 (TNFR1) and/or interleukin receptor (TLR) on the cell membrane of lung fibroblasts, so that the presence of NF-κB and inhibitor IκBα in the cytoplasm form a trimer, and the activated NF-κB is transferred to the nucleus to regulate the transcription of target genes and output signals (36, 37). Currently, the NF-κB signaling pathway is activated.

**Fig. 8:**
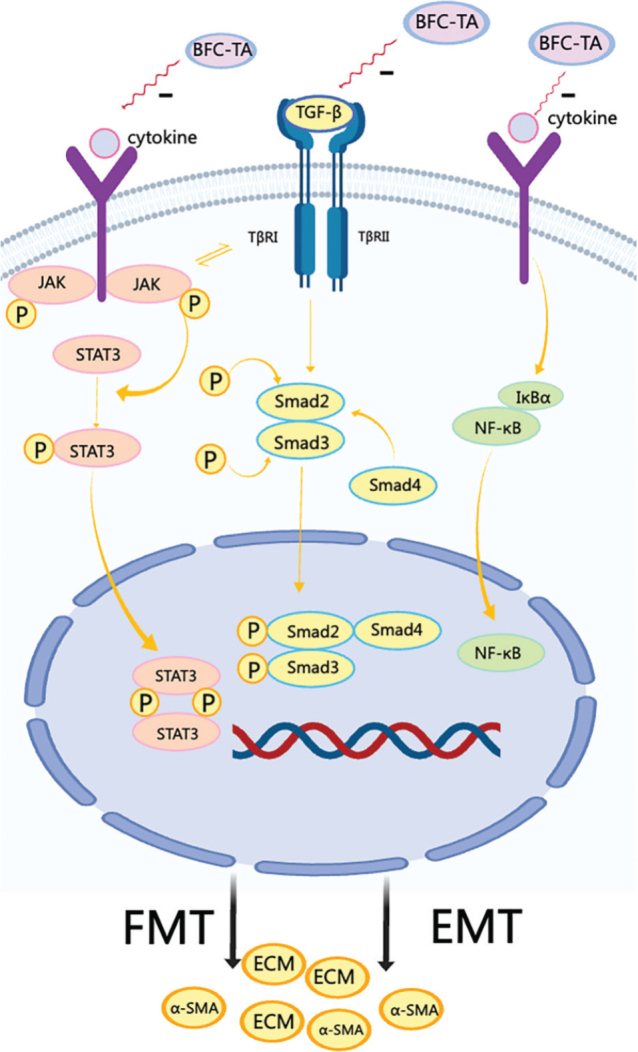
The mechanism of transforming growth factor-β (TGF-β) signaling pathway and NF-κB signaling pathway

EMT describes the biological process through which epithelial cells undergo particular procedures to change into cells with a mesenchymal phenotype. It is crucial for the development of the embryo, chronic inflammation, cancer metastasis, and a number of fibrotic illnesses (38, 39). The main characteristic markers of this process are fibronectin and E-cadherin. EMT process promotes the release of fibronectin and inhibits the release of E-cadherin. Activation of TGF-β-related signaling pathways can induce EMT process, promote the release of Fibronectin and other proteins, and inhibit the release of E-cadherin and other proteins (40). In addition, the activation of TGF-β-related signaling pathways can lead to the transformation of lung fibroblasts into lung myofibroblasts (FMT process) (41). The EMT and FMT process also secrete a large amount of α-smooth muscle actin (markers of fibroblast activation) and ECM, such as collagen (42, 43). When α-SMA is overexpressed, ECM is oversecreted and deposited (44) (Fig. 8). Our study found that BFC-TA treatment reversed the expression of the EMT process, increased the release of E-cadherin in BLM-induced rat lung tissue, reduced the release of fibronectin, and also reduced the levels of Collagen I, α-SMA, and HYP. expression. Treatment with BFC-TA also significantly reduced the activation of the NF-κB signaling pathway and the expression of related inflammatory responses, as well as the expression of the TGF-β/Smad and STAT3 signaling pathways. These results indicate that BFC-TA inhibits the progression of pulmonary fibrosis by inhibiting the EMT process and NF-κB and TGF-β related signaling pathways.

Pirfenidone is the first drug to obtain marketing approval for the treatment of idiopathic pulmonary fibrosis (45). It is a commonly used drug in clinical practice, which can effectively inhibit the activation of pulmonary fibrosis cells, improve lung function, and delay the progression of pulmonary fibrosis (46). However, pirfenidone has certain side effects, such as digestive tract diseases, liver function impairment, etc. (47). The results of this study found that when BFC-TA treats pulmonary fibrosis through the TGF-β-related signaling pathway, the therapeutic effect of the BFC-TA high-dose group (136.mg/kg) is close to the therapeutic effect of the PFD positive control (150 mg/kg), even better than the PFD group. We speculate that bulbus of *Fritillaria cirrhosa* is a plant with the same origin as medicine and food, with few side effects. The composition of total alkaloids is diverse and complex, and it has the potential to treat pulmonary fibrosis with multiple targets. Therefore, it has the potential to be developed as a preventive health food for pulmonary fibrosis and a new drug for the treatment of pulmonary fibrosis, providing a theoretical basis for clinical research.

## Conclusion

This study explored the effect of BFC-TA on bleomycin-induced pulmonary fibrosis in rats. The results showed that BFC-TA can effectively delay BLM-induced pulmonary fibrosis. This study showed that 136.8 mg/kg BFC-TA has the best effect in treating BLM-induced pulmonary fibrosis, even better than PFD (150 mg/kg). BFC-TA can reduce the inflammatory response, the activation of lung fibroblasts, and collagen and fiber deposition, which plays an antifibrosis role (Fig. S1). Therefore, this study observed that BFC-TA is a potential ingredient that delays the progression of pulmonary fibrosis, providing a reference for the creation of health foods for the treatment of pulmonary fibrosis .

## Authors’ contribution

Implementing the research process, Formal analysis, Writing-original draft: Mingxin Pai; Writing review and editing: AGA Er-bu; Researching and organizing literature, Drawing legend: Yexin Wu; Supervision, Project administration: Tse Wai Ming; Resources: Tse Kathy Wai Gaun; Writing review and editing, Supervision, Project administration, Funding acquisition: Bengui Ye.

## Conflict of interest and funding

The authors declare no conflicts of interest. This research was funded by Major science and technology research project in 2021 from Tibet Science and Technology Department, Research on the product transformation of Tibetan genuine medicinal material (Tibetan Fritillaria Bulb) in the treatment of the chronic obstructive pulmonary disease (COPD) (XZ202101ZD0021G), Science and Technology Major Project of Tibetan Autonomous Region of China (XZ202201ZD0001G01; XZ202201ZD0001G06), Funds for local scientific and technological development guided by the central government in 2023 from Tibet Science and Technology Department (XZ202301YD0014C), Major science and technology research project in 2023 from Tibet Science and Technology Department (XZ202301ZY0009G).
