# Supplementary material for: Total alkaloids of bulbus of Fritillaria cirrhosa alleviate bleomycin-induced inflammation and pulmonary fibrosis in rats by inhibiting TGF-β and NF-κB signaling pathway
Source: Food Nutr Res. 2023 Dec 29;67:10.29219/fnr.v67.10292. doi: 10.29219/fnr.v67.10292 (PMC10770707; doi:10.29219/fnr.v67.10292)
Supplement: Supplementary file 1 [file FNR-67-10292-s001.pdf]

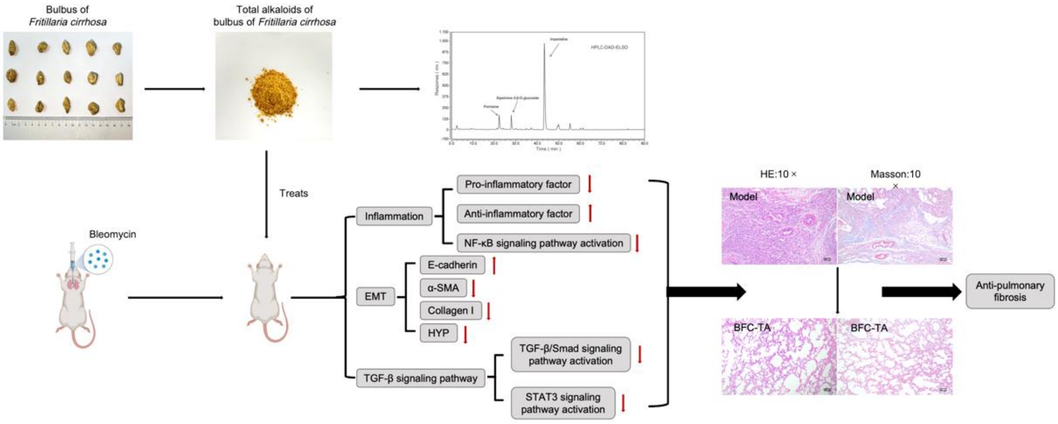

Fig.S1 Graphical Abstract of this article

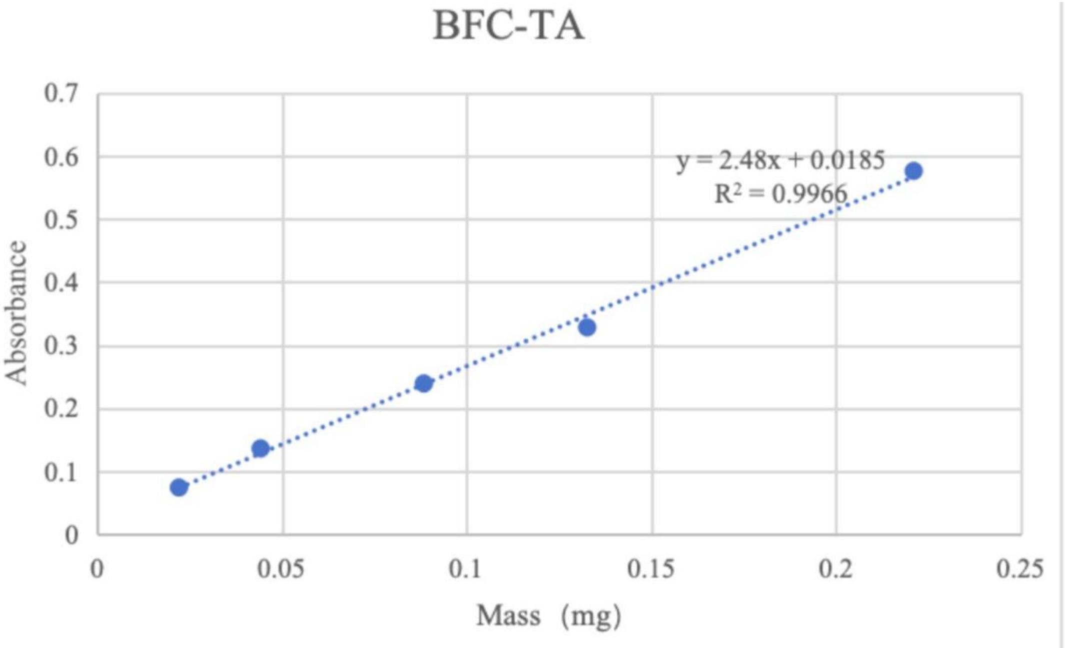

Fig.S2 Standard curve of Imperialine by ultraviolet spectrophotometry for determining the content of BFC-TA

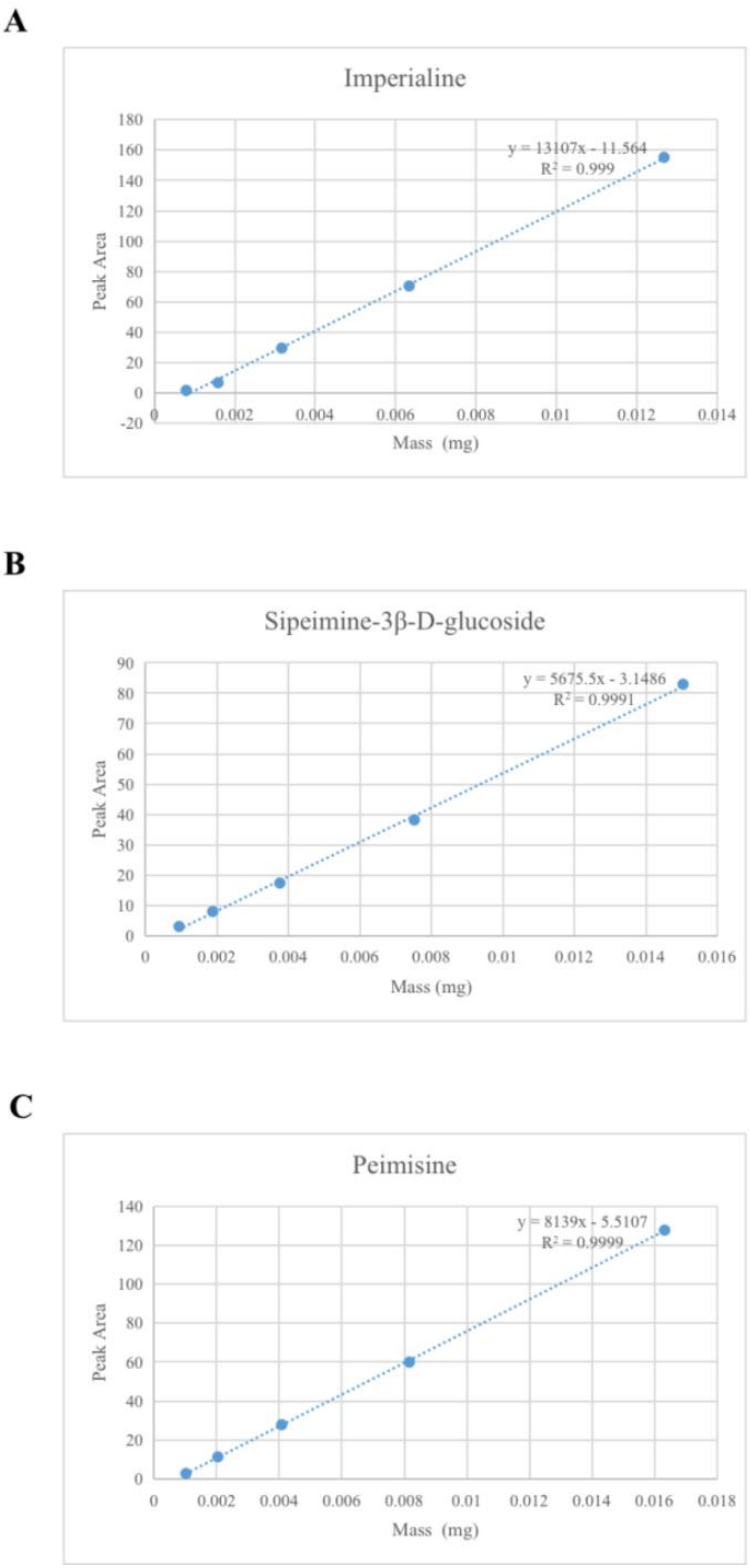

Fig.S3 Standard curves of Imperialine, Sipeimine-3β-D-glucoside and peimisine were prepared by HPLC-DAD-ELSD

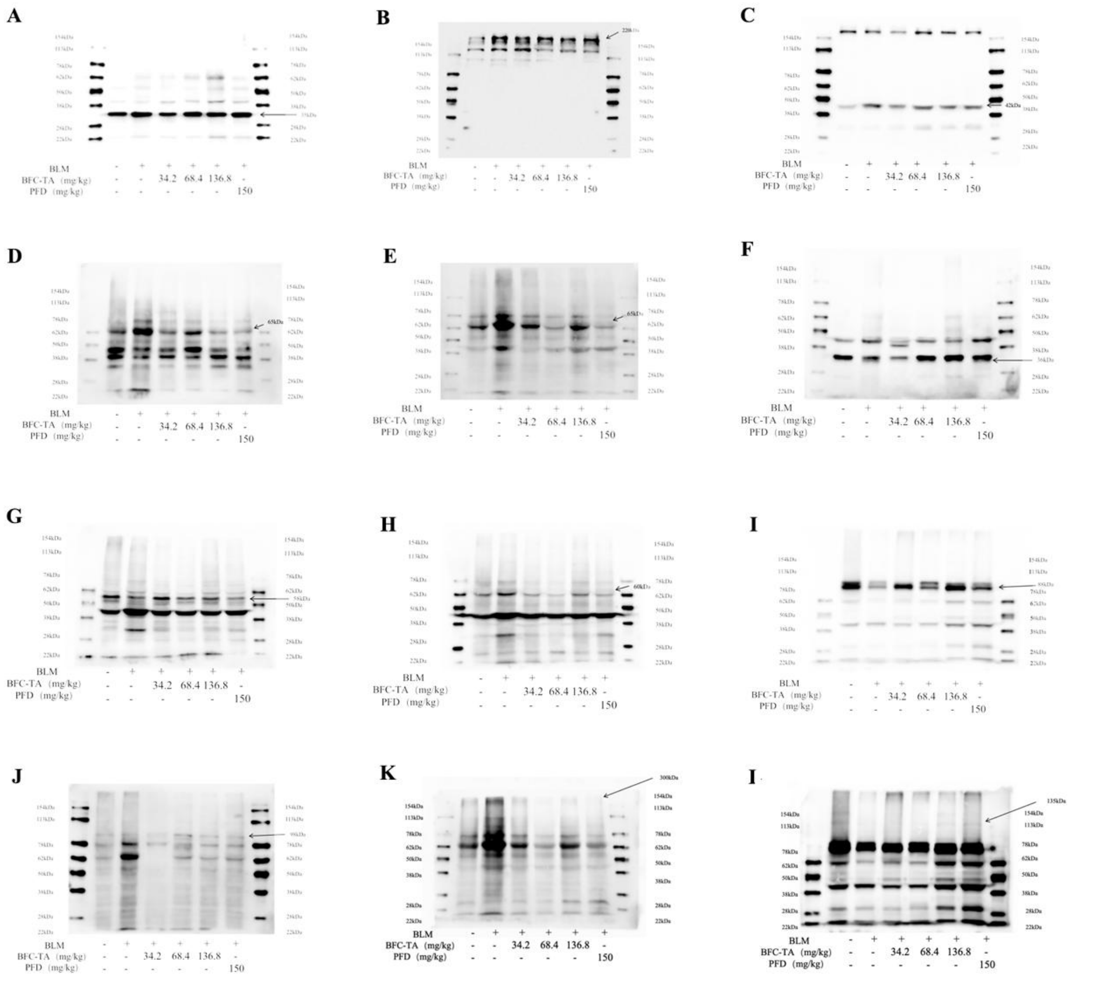

Fig.S4 (A) Whole membrane of GAPDH, protein molecular weight is 35kDa; (B) Whole membrane of Collagen I, protein molecular weight is 220kDa; (C) Whole membrane of α-SMA, protein molecular weight is 42kDa; (D) Whole membrane of NF-κB, protein molecular weight is 65kDa; (E) Whole membrane of p-NF-κB, protein molecular weight is 65kDa; (F) Whole membrane of IκBα, protein molecular weight is 36kDa; (G) Whole membrane of Smad2, protein molecular weight is 58kDa; (H) Whole membrane of p-Smad2, protein molecular weight is 60kDa; (I) Whole membrane of STAT3, protein molecular weight is 88kDa; (J) Whole membrane of p-STAT3, protein molecular weight is 98kDa; (K) Whole membrane of Fibronectin,

protein molecular weight is 300kDa; (L) Whole membrane of E-Cadherin, protein molecular weight is 65kDa. This membrane is obtained by washing the whole membrane of STAT3 and then incubating it with Fibronectin primary antibody.
